# The effect of hippocampal function, volume and connectivity on posterior cingulate cortex functioning during episodic memory fMRI in mild cognitive impairment

**DOI:** 10.1007/s00330-017-4768-1

**Published:** 2017-03-13

**Authors:** Janne M. Papma, Marion Smits, Marius de Groot, Francesco U. Mattace Raso, Aad van der Lugt, Henri A. Vrooman, Wiro J. Niessen, Peter J. Koudstaal, John C. van Swieten, Frederik M. van der Veen, Niels D. Prins

**Affiliations:** 1 Department of Neurology, Erasmus MC – University Medical Center Rotterdam, ’s‐Gravendijkwal 230, 3015 CE Rotterdam, The Netherlands; 2 Department of Radiology, Erasmus MC – University Medical Center Rotterdam, Rotterdam, The Netherlands; 3 Department of Medical Informatics, Erasmus MC – University Medical Center Rotterdam, Rotterdam, The Netherlands; 4 Department of Geriatrics, Erasmus MC – University Medical Center Rotterdam, Rotterdam, The Netherlands; 5 Faculty of Applied Sciences, Delft University of Technology, Delft, The Netherlands; 6 Institute of Psychology, Erasmus University Rotterdam, Rotterdam, The Netherlands; 7 Alzheimer Center, Department of Neurology, VU University Medical Center, Neuroscience Campus, Amsterdam, The Netherlands

**Keywords:** Mild cognitive impairment, Episodic memory task-related functional MRI, Diffusion tensor imaging, Posterior cingulate cortex, Hippocampus

## Abstract

**Objectives:**

Diminished function of the posterior cingulate cortex (PCC) is a typical finding in early Alzheimer’s disease (AD). It is hypothesized that in early stage AD, PCC functioning relates to or reflects hippocampal dysfunction or atrophy. The aim of this study was to examine the relationship between hippocampus function, volume and structural connectivity, and PCC activation during an episodic memory task-related fMRI study in mild cognitive impairment (MCI).

**Method:**

MCI patients (n = 27) underwent episodic memory task-related fMRI, 3D-T1w MRI, 2D T2-FLAIR MRI and diffusion tensor imaging. Stepwise linear regression analysis was performed to examine the relationship between PCC activation and hippocampal activation, hippocampal volume and diffusion measures within the cingulum along the hippocampus.

**Results:**

We found a significant relationship between PCC and hippocampus activation during successful episodic memory encoding and correct recognition in MCI patients. We found no relationship between the PCC and structural hippocampal predictors.

**Conclusions:**

Our results indicate a relationship between PCC and hippocampus activation during episodic memory engagement in MCI. This may suggest that during episodic memory, functional network deterioration is the most important predictor of PCC functioning in MCI.

***Key Points*:**

• *PCC functioning during episodic memory relates to hippocampal functioning in MCI*.

• *PCC functioning during episodic memory does not relate to hippocampal structure in MCI*.

• *Functional network changes are an important predictor of PCC functioning in MCI*.

**Electronic supplementary material:**

The online version of this article (doi:10.1007/s00330-017-4768-1) contains supplementary material, which is available to authorized users.

## Introduction

Mild cognitive impairment (MCI) is a clinical construct that identifies individuals with cognitive impairment and a high risk of dementia [1–3]. Although MCI is a heterogeneous condition, most MCI patients exhibit the amnestic phenotype with episodic memory deficits as the sole or most prominent characteristic [4]. In those patients, Alzheimer’s disease (AD) is the most common clinical endpoint [1, 5], and the amnestic deficits are considered to be the consequence of neuropathological changes affecting the medial temporal lobe (MTL) early in the disease process. While structural and functional MRI studies concerning memory functioning in MCI and AD, initially focused on the MTL or specifically hippocampus [6], current neuroimaging studies examine patterns of deterioration in global functional and structural brain circuits or networks, e.g. the Papez circuit [7, 8], or the more recently described default mode network (DMN) [9–12]. Within these networks though, the effects of medial temporal lobe degeneration on network functioning, or functioning of specific network nodes, is still unclear [8, 13].

The posterior cingulate cortex (PCC) is an important network node, showing hypometabolism and hypoperfusion in the MCI stage [14–19], predictive for further cognitive decline into clinical AD [14, 20, 21]. Given its network connection with the hippocampus, several studies debated whether changes in PCC functioning might reflect deterioration of the hippocampus in early disease stages [13, 20, 22–30]. From these studies we can grossly deduce three theories, namely that PCC functioning in MCI might reflect: (1) functional changes in the hippocampus [9, 24, 31–33]; (2) structural changes in the hippocampus [13, 14, 24–28]; or (3) degeneration of white matter tracts subserving a part of the connection between the hippocampus and PCC [29, 30, 34–36]. It is known that the hippocampus has multiple efferent connections, and communication between the hippocampus and PCC also runs through the thalamus [8, 22], or several other nodes, as the functional path length between the hippocampus and PCC is shown to alter in MCI in relationship with cognitive decline [37]. Meanwhile, there are a number of clinical studies that have specifically indicated cingulum disruption to subserve memory problems due to a disconnection between the hippocampus and PCC [34, 38–40]. As the PCC is a key hub, and PCC dysfunctioning is shown to be an indicator for AD prodromal stages, examining the relationship between PCC functioning and hippocampus functioning, hippocampus structure and structural connectivity in MCI will provide further insight into the origin of PCC dysfunctioning in MCI. In this multimodal MRI study we performed analyses on the association between PCC functioning during task-related episodic memory fMRI and (1) hippocampus activation (episodic memory fMRI), (2) hippocampus volume (automatic segmentation of hippocampus), and (3) structural connections subserving partly the connection between the hippocampus and PCC (diffusion tensor imaging; DTI), in patients with MCI.

## Material and methods

### Participants

Thirty-three MCI patients underwent episodic memory task-related fMRI, structural MRI, DTI and extensive neuropsychological assessment. MCI patients were recruited from 2009–2011 on the basis of the criteria of Petersen [4], as criteria for MCI due to AD by Albert et al. [1] were not published at time of recruitment. Exclusion criteria for all participants were: history of neurological or psychiatric disorders negatively affecting cognition (e.g. major stroke, cerebral tumour or depression) and contraindication for MRI (e.g. metal implants, claustrophobia).

All participants gave informed consent to the protocol of the study, which was approved by the medical ethics committee of the Erasmus MC. On the basis of fMRI task performance (as specified below) or movement during scanning, we excluded six MCI patients, and thus eventually included 27 MCI patients in our analyses.

### Structured interview

Data on demographics were collected during a structured interview. We assessed level of education by means of a Dutch education scale, with a range from 1 (less than 6 years of elementary school) to 7 (academic degree) [41]. We administered the Mini-Mental State Examination (MMSE) as a global cognitive screening method.

### Neuropsychological assessment

All participants underwent an extensive neuropsychological assessment. For every neuropsychological test, we calculated z-scores using the mean and standard deviation from a control group of 15 healthy elderly (mean age 70.6 years, mean MMSE 28.9), and subsequently constructed compound scores for cognitive domains (see Supplementary Material and Methods).

### MRI acquisition protocol

We performed functional and structural MR imaging on a 3.0T MRI scanner with an 8-channel head coil (HD platform, GE Healthcare, Milwaukee, WI, US). All participants underwent an MRI protocol including 3D T1w MRI, 2D T2-FLAIR, DTI and fMRI during an episodic memory task. Details on MRI sequence parameters can be found in the Supplementary Material and Methods.

### Automated MRI brain tissue segmentation and volumetric analysis of hippocampi

We automatically classified brain tissue on MRI into cerebrospinal fluid, grey matter, normal appearing white matter, white matter hyperintensities (WMH), based on intensities of the 3D T1-weighted and 2D T2-FLAIR MRI scans [42–44]. We segmented the hippocampus using the 3D T1-weighted image by means of an automated method as described previously [45, 46]. Blinded for clinical information, we visually inspected the results of all automated segmentations, and if necessary (one case), the automated segmentations were manually corrected using FSLview as part of FSL [45]. An experienced neurologist (NDP) and neuroradiologist (MS) visually evaluated the occurrence and location of thalamic lacunar infarcts on the basis of 2D T2-FLAIR, though we acknowledge the low sensitivity of T2-FLAIR for the detection of thalamic lesions [47].

### DTI data processing and probabilistic tractography

We applied an extensive diffusion tensor imaging (DTI) data processing method, in which we used the eigenvalues of the fitted diffusion tensors to provide measures of fractional anisotropy (FA), mean diffusivity (MD), axial diffusivity (AxD) and radial diffusivity (RD). These measures and methods are described in detail in a study by Papma et al. [42]. To rule out the effects of vascular-related macrostructural white matter damage in the DTI data, we restricted DTI analyses to the normal appearing white matter by mapping the individual WMH segmentation masks on the DTI maps and excluding voxels originating from WMH from our analyses, as described previously [42]. We performed automated probabilistic tractography for the cingulum along the hippocampus (CGH). These analyses were performed in subject native space by means of Probtrackx, available in FSL. We used standard space seed, target, stop and exclusion masks, placed in accordance with the protocols described by Wakana et al. [48, 49]. To adopt the region from Wakana et al. for probabilistic tractography, additional masks were included to prevent an over segmentation of the CGH (also see [50]). Seed and target masks are slightly larger than the masks indicated in Wakana, in order to be robust against slight misalignment introduced in the registration step of the tractography. Further information on our approach is publicly available on the FSL website (http://fsl.fmrib.ox.ac.uk/fsl/fslwiki/AutoPtx). The masks were transferred to subject-native space using nonlinear registration obtained with default settings for FA images in FNIRT. The tract density image for each tract was normalized by division with the total number of fibre paths recorded in the tract density image. These images were then thresholded at 0.005 to yield binary segmentations.

### Functional MRI paradigm

We engaged episodic memory by means of an event-related verbal episodic memory task including separate encoding and recognition runs, based on the fMRI paradigm of Daselaar et al. [51]. Verbal stimuli consisted of emotionally neutral words, visually presented to participants. During encoding, participants were asked to indicate a positive or negative emotion related to the presented stimulus in order to improve consolidation, by means of respectively a right-or left-hand button press. Words presented in the recognition run consisted of words presented during the encoding run, intermixed with new words. Participants were asked to indicate whether they recognized a word or not by means of respectively a right or left-hand button press. Both the encoding and recognition runs included baseline stimuli consisting of the words ‘left’, indicating a left-hand press, and ‘right’, indicating a right-hand press. Stimuli that elicited no reaction were excluded from further analyses. Details on the task design can be found in the Supplementary Material and Methods.

### Functional MRI in-scanner task performance

If participants correctly recognized a stimulus, this was considered ‘correct recognition’ in the recognition run, and consequently ‘successful encoding’ in the encoding run. We examined task performance by calculating d prime, a measure indicating the ability to discriminate between target and distractor words in episodic memory tasks [52]. D prime was calculated as (Z-score false alarm) minus (Zscore correct recognition). Lower d prime scores reflect lower sensitivity in discriminating target from distractor items. Participants were excluded from the study if their task performance during recognition was equal to or below chance level, if the number of missed stimuli exceeded the number of correct recognition stimuli, or if the number of false alarm stimuli exceeded the number of correct rejections. This was done in order to be able to attribute functional MRI changes to potential neurodegenerative processes rather than task performance [53].

### Functional MRI data analysis

We analysed fMRI data using Statistical Parametric Mapping software (SPM8; Wellcome Department of Cognitive Neurology, London, UK) implemented in Matlab R2013b (Mathworks, Natick, MA, USA). Details on data preprocessing in SPM can be found in the Supplementary Material and Methods. For encoding and recognition analyses we used separate design matrices. In the encoding design matrix we included baseline stimuli and successfully encoded stimuli as separate parameters, as well as realignment parameters. In a first level analysis we created individual ‘successful encoding versus baseline’ contrast maps. These contrast maps were entered in a second level analysis where we performed an activation and deactivation within group analysis. In the design matrix for the recognition phase we entered regressors for baseline stimuli and correct recognition. In a first level analysis we created the ‘correct recognition versus baseline’ contrast on an individual level, and subsequently entered the contrast maps in a second level analysis where we performed an activation and deactivation within-group analysis. Significance was tested at p <0.05 with family wise error (FWE) correction for multiple comparisons, as well as at a more lenient threshold of p < 0.001 not corrected for multiple comparisons, and a cluster size of at least 20 voxels. In a post hoc analysis we added a covariate for MMSE, to test to what extent the severity of disease influences results. Anatomical labelling of significantly activated clusters was performed using WFU Pickatlas software extension to SPM8 (Functional MRI laboratory - Wake Forest University School of Medicine, Winston Salem). A priori defined regions of interest (ROIs) of the bilateral PCC, left and right hippocampus, and left and right parahippocampal gyrus were created using AAL masks. Within these ROIs we extracted mean beta values for successful encoding and correct recognition using Marsbar, and subsequently exported these to SPSS (version 21.0 for Windows) for stepwise linear regression analyses.

### Statistical analysis

Demographic and neuropsychological data was analysed using SPSS version 21.0 for Windows. To determine whether (1) hippocampus activation, (2) volume or (3) white matter connectivity was associated with PCC activation during successful episodic memory encoding and correct recognition in MCI, we performed a stepwise linear regression analysis with the PCC beta values during successful encoding and correct recognition as dependent variable and the independent variables: beta values of left and right hippocampus (respectively during successful encoding and correct recognition), left and right hippocampal volume in ml, mean FA, MD, AxD and RD of the left and right CGH. Post hoc, we performed the same analysis, but added the left and right parahippocampal gyrus activation to the independent variables. To reduce the risk of overfitting associated with a stepwise regression analysis, we performed a Bonferroni adjustment for the analyses [54].

## Results

### Participant characteristics, neuropsychological profile and MRI characteristics

Demographic, neuropsychological and MRI characteristics of MCI patients are displayed in Tables 1, 2 and 3. As expected, MCI patients had lowered Z-scores on neuropsychological tests for immediate and delayed memory recall (>2 SD for delayed recall, Table 2), but also performed worse on tests for executive function and language than controls (Table 2). One MCI patient showed a lacunar infarct in the lateral right thalamus.

**Table 1:** Demographic characteristics of mild cognitive impairment (MCI) patients

	MCI (n = 27)
Age, years	73.9 (4.9)
Sex, women (%)	5 (18.5)
Education	5.6 (1.0)
MMSE	27.5 (2.0)

Values are unadjusted means (standard deviation) or number of participants (percentages)

*MMSE* Mini-Mental State Examination

**Table 2:** Neuropsychological assessment and fMRI performance of mild cognitive impairment (MCI) patients

	MCI (n = 27)
Memory immediate recall	−1.39 (0.66)
Memory delayed recall	−2.49 (2.15)
Processing speed	−0.46 (0.94)
Executive functioning	−1.14 (1.85)
Language	−0.83 (1.39)
Visuospatial ability	0.04 (1.42)
Visuoconstructive ability	−0.17 (1.85)
D prime episodic memory during fMRI	1.69 (0.71)

Neuropsychological results displayed as mean z-scores for cognitive domains (standard deviation), relative to a group of n = 15 controls

*fMRI* functional MRI

**Table 3:** Neuroimaging results

	MCI (n = 27)
Left hippocampal volume in ml	2.8 (0.5)
Right hippocampal volume in ml	2.9 (0.4)
Grey matter volume in ml	457.4 (68.5)
White matter volume in ml	412.8 (52.2)
White matter hyperintensity volume in ml (log)^1^	3.2 (2.8-3.6)
Left FA CGH	0.36 (0.03)
Left MD CGH^2^	1.05 (0.09)
Left AxD CGH^2^	1.47 (0.13)
Left RD CGH^2^	0.84 (0.08)
Right FA CGH	0.39 (0.03)
Right MD CGH^2^	1.04 (0.09)
Right AxD CGH^2^	1.49 (0.11)
Right RD CGH^2^	0.82 (0.08)
Beta values PCC during successful encoding	-1.16 (2.10)
Beta values left hippocampus during successful encoding	-0.08 (1.71)
Beta values right hippocampus during successful encoding	-0.22 (1.36)
Beta values PCC during correct recognition	-0.39 (1.68)
Beta values left hippocampus during correct recognition	0.07 (1.53)
Beta values right hippocampus during correct recognition	0.08 (1.04)

Values are unadjusted means (standard deviation)

^1^ median (interquartile range)

^2^ MD, AxD and Rd shown as 10^-3^ mm^2^/s. In case of median testing the Mann Whitney U-test was used

*MCI* mild cognitive impairment, *FA* fractional anisotropy, *MD* mean diffusivity, *AxD* axial diffusivity, *RD* radial diffusivity, *CGH* cingulate along the hippocampus, *PCC* posterior cingulate cortex

### Functional MRI results

**Fig. 1:**
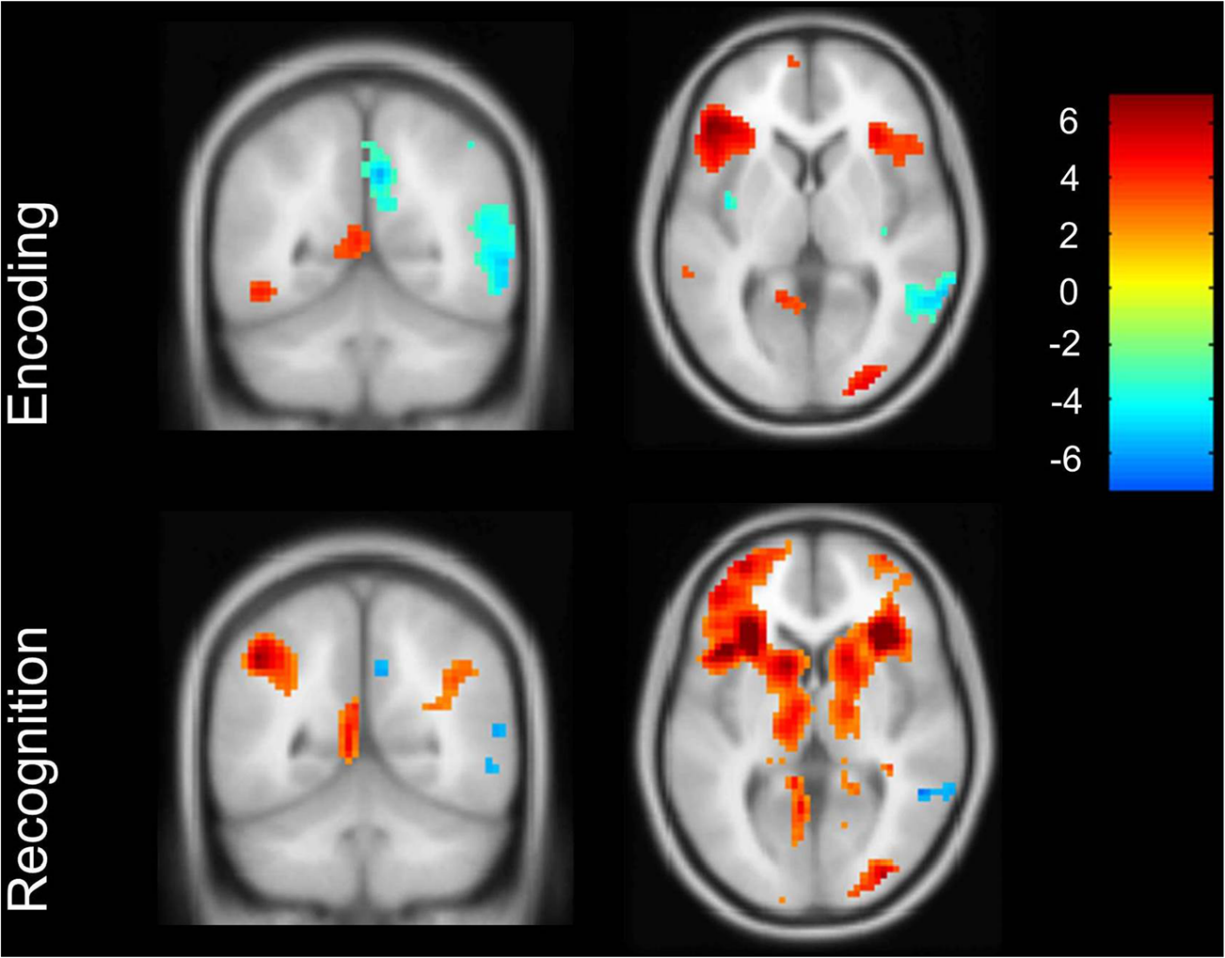
Activation and deactivation patterns in successful encoding versus baseline and correct recognition versus baseline, lenient threshold without correction for multiple comparisons. Coronal view in posterior cingulate cortex (PCC) region

**Fig. 2:**
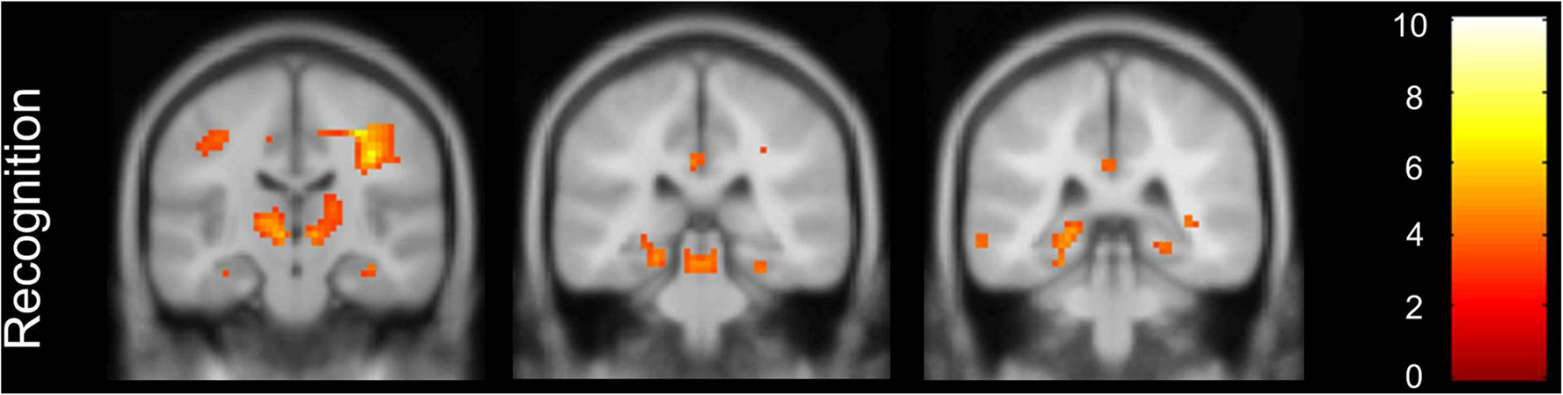
Activation of the hippocampus extending into the parahippocampal gyrus in mild cognitive impairement (MCI) patients during recognition, lenient threshold without multiple comparisons

Within group activation analyses for memory encoding at a threshold of p <0.05 with FWE correction for multiple comparisons, showed left inferior and medial frontal gyrus and left cingulate gyrus activation for MCI patients. The activation pattern was extended to the right hemisphere when using a more lenient threshold of p < 0.001, without correction for multiple comparisons (Fig. 1, Supplementary Results Table 1) and included the bilateral medial and inferior frontal gyrus, the left middle temporal gyrus, the left PCC, the left postcentral gyrus and the bilateral inferior occipital gyrus. Within-group deactivation analyses for memory encoding showed no deactivation at a threshold of *p* < 0.05 with FWE correction for multiple comparisons, and right postcentral gyrus, bilateral inferior parietal gyrus, right precuneus, right PCC and the right mid cingulate gyrus deactivation at a lenient threshold (Fig. 1, Supplementary Results Table 1). Upon visual inspection, the recognition memory activation pattern was more extensive, with activation in the right inferior frontal gyrus, the left insula, the bilateral middle and right inferior occipital gyrus, the bilateral inferior and right superior parietal gyrus (Fig. 1, Supplementary Results Table 1), however lacking PCC activation (Fig. 1). During recognition we found hippocampal and parahippocampal gyrus activation in MCI patients, in clusters smaller than 20 voxels, displayed in Fig. 2. Within-group deactivation during recognition was found in the middle temporal gyrus (Fig. 1, Supplementary Results Table 1). In a post hoc analysis we added the MMSE as a covariate. Results were similar to the analyses without this covariate (see Supplementary Results Table 2).

### Stepwise linear regression analyses of PCC

Table 4 summarizes the results for the stepwise regression model. A model with right hippocampal activation was significantly associated with PCC activation during successful encoding, while left hippocampal activation was significantly related to PCC activation during correct recognition (Table 4). We performed a post hoc analysis in which we also included the left and right parahippocampal gyrus activation respectively during encoding and recognition. In this second stepwise regression analysis, the right and left hippocampus remained the most important predictors for PCC activation during respectively encoding and recognition. The p values associated with left and right parahippocampal gyrus volumes in the model of PCC activation during encoding were left parahippocampal gyrus: (T 0.334) p = 0.741, and right parahippocampal gyrus: (T 0.586) p = 0.618; and during recognition left parahippocampal gyrus: (T 0.555) p = 0.584, and right parahippocampal gyrus: (T 1.493) p = 0.149.

**Table 4:** Stepwise regression model in mild cognitive impairment (MCI) patients

Dependent variable	Predictors	Beta value	Sig	R^2^
PCC beta values encoding	1. Right hippocampus beta values encoding	0.960	0.0005	0.390
PCC beta values recognition	1. Left hippocampus beta values recognition	0.853	<0.0001	0.607

Significant predictors in a stepwise regression model. Beta values are unstandardized coefficients. Bonferroni corrected threshold of p < 0.0041

*PCC* posterior cingulate cortex

## Discussion

Our results indicate an association between PCC activation and hippocampus activation during successful episodic memory encoding and correct recognition in MCI patients. We found no relationship between structural hippocampal predictors, such as the CGH subserving a part of the connection between the PCC and hippocampus or hippocampal volumes, and PCC activation. While this suggests that episodic memory decline in MCI can best be explained as a functional network disorder, structural and functional connectivity between the PCC and hippocampus is known to be quite complex in MCI and AD [8, 37].

The finding of a relationship between hippocampal and PCC activation in the present study is supported by several functional neuroimaging studies in healthy individuals, MCI or dementia patients [31, 55–57]. The absence of a relationship between structural hippocampal measures and PCC activation though, contrasts other studies. In these studies hippocampal volume was related to either perfusion within the PCC [13, 25], fMRI task-induced PCC deactivation [58] or functional connectivity between both structures [27]. One reason for the difference in findings may be methodological, as our study was performed in an episodic memory-related fMRI setting, which enables us to investigate hippocampal and PCC activation while these structures are engaged in episodic memory. From the current clinical literature we can grossly deduce three theories that have been examined in previous studies, concerning the effects of disease-related changes in the hippocampus on PCC functioning. First, a theory concerning a relationship between PCC dysfunctioning and hippocampal grey matter atrophy via the degeneration of white matter bundles that subserve a part of the connections between the hippocampus and PCC [26, 38, 59]. This is supported by several studies showing a relationship between hippocampal volume and PCC metabolism mediated through cingulum bundle disruption [26, 59, 60]. Second, the theory that independent white matter tract degeneration disconnects the hippocampus from other structures, and subsequently affects episodic memory [29, 30, 36, 39, 40, 61]. Third, a theory concerning the deterioration of functional networks, in which brain regions synchronically function less well [12, 55]. As we found a relationship between hippocampal and PCC activation, our results can only support the functional network deterioration theory. However, we acknowledge that the structural and functional connectivity between the PCC and hippocampus subserving episodic memory in MCI is more complex [8, 37].

We found that the most important predictor of PCC activation during successful episodic memory encoding was right hippocampal activation, whereas left hippocampal activation was a significant predictor for PCC functioning during episodic memory recognition. This pattern of hemispheric specialization for different phases of episodic memory has been presented in the literature, with right temporal lobe dysfunction having the greatest effect on acquisition of new learning, and left temporal lobe dysfunction on retention or retrieval [62, 63]. In particular in case of verbal episodic memory engagement it was shown that other regions can be involved besides the PCC and hippocampus, like the medial and inferior frontal gyrus and the insula, and as stated below, the thalamus [32, 64–67].

Increasing evidence suggests that while the hippocampus is an important node in memory functioning, other nodes within the mnemonic system or Papez circuit or DMN can also lead to memory dysfunctioning [8]. As expected, the PCC is one of these nodes, but recent evidence shows that thalamus functioning is critical in memory functioning [8]. With its numerous connections as a relay system, the thalamus subserves connections between the hippocampus and PCC. Through this interdependent relationship with the PCC, early thalamus damage or dysfunction may also be explanatory for the earliest PCC imaging evidence of early AD [8]. In the current study one MCI patients showed a thalamus infarct in light of relatively mild medial temporal lobe atrophy (left MTA 1, right MTA 2) as evaluated on 2D T2 FLAIR. We acknowledge the limitations of T2 FLAIR to detect thalamic lesions [47], and the possibility of underestimating the number and the role of thalamic lesions in the current study. Therefore, future studies should include (1) T2-weighted images in order to examine thalamic lesions, and (2) evaluate anterior thalamic functioning when elucidating influence on PCC functioning during memory tasks in MCI.

In this study we cannot rule out a direct relationship between PCC function and neuropathological changes within this region. One study suggested that amyloid burden disrupts functional networks in healthy elderly [33]; however, PCC amyloid deposition in MCI was not found to be related to functional changes within the PCC region [68] nor to clinical status or AD disease progression [69–73]. Furthermore, the PCC region is relatively unaffected by neurofibrillary tangle (NFT) formation in early AD disease stages [74, 75]. Hippocampal atrophy, on the other hand, is related to NFT formation [76], directly reflected in episodic memory impairment at the MCI stage. As one study claims that PCC volume loss and cingulum bundle deterioration secondary to MTL atrophy both influence PCC functioning [60], the role of PCC degeneration in the face of relative late NFT formation [74, 75] could be obtained as a sequential process of functional decline followed by structural decline later in the disease process.

Strengths of our study are the use of an event-related fMRI design that enabled us to investigate PCC and hippocampus functioning during episodic memory component processes such as successful encoding and correct recognition. A drawback of the current study is the inability to use the research criteria for MCI due to AD in the present study [1], as our participants were included in the years 2009–2011, before these criteria were established. All MCI patients though presented with amnestic MCI, which is known to have a high rate of conversion to clinical AD [77–79]. We did not include AD biomarkers at the time, since PiB-PET was still in its infancy, and lumbar puncture was considered too invasive by our medical ethics committee for patients in a pre- or early clinical stage of dementia. Furthermore, although the number of MCI patients in this study was comparable to other studies using memory-related fMRI in MCI [64], our study may suffer from a lack of power. This could explain the fact that hippocampus and PCC activation or deactivation in both the encoding and recognition tasks was minimal. This is an important limitation of our study, in which a priori hypotheses considered the relationship between PCC and hippocampus activation.

In the present study, we found a relationship between activation in the PCC and hippocampus during successful episodic memory encoding and correct recognition in MCI. We found no evidence for the influence of often referred structural hippocampal predictors, on PCC functioning. Our results suggest that in MCI, PCC functioning is foremost influenced by hippocampal functioning during episodic memory, reflecting possibly a pattern in which functional changes precede or exceed structural changes.

## Electronic supplementary material

Below is the link to the electronic supplementary material.

ESM 1 (DOCX 134 kb)
