# Supplementary material for: The effect of hippocampal function, volume and connectivity on posterior cingulate cortex functioning during episodic memory fMRI in mild cognitive impairment
Source: Eur Radiol. 2017 Mar 13;27(9):3716–24. doi: 10.1007/s00330-017-4768-1 (PMC5544779; doi:10.1007/s00330-017-4768-1)
Supplement: Supplementary file 1 — (DOCX 134 kb) [file 330_2017_4768_MOESM1_ESM.docx]

**SUPPLEMENTARY MATERIAL and METHODS**

*Neuropsychological assessment*

The compound score for memory comprised the z-scores of the Dutch version of the Rey Auditory 15-word verbal learning test (15-WVLT), immediate and delayed recall and the stories of the Rivermead Behavioural Memory Test (RBMT), immediate and delayed recall. The compound score for mental processing speed comprised z-scores of the Trail Making Test (TMT) part A and Stroop II. Executive functioning compound scores were calculated combining z-scores of the TMT part B and Stroop III. The compound score for language (word finding difficulties and lexical retrieval) comprised z-scores of the Boston Naming Test (BNT; 60 items version) and semantic fluency tasks (animals and occupations). The subtest Block Design of the revised Wechsler Adult Intelligence Scale (WAIS-R) and clock drawing were used to assess visuospatial and visuoconstructive ability respectively, z-scores for these domains were calculated using these single tests.

*MRI sequence parameters*

High resolution 3D T1-weighted structural MRI was acquired in the axial plane with the following parameters: repetition time (TR) = 10.4 ms, echo time (TE) = 2.1 ms, inversion time (TI) = 300 ms, flip angle = 18°, acquisition matrix = 416x256, field of view (FOV) = 250x175 mm^2^. 192 slices were acquired with a slice thickness of 1.6 mm with 0.8 mm overlap, resulting in an effective slice thickness of 0.8 mm, in a total acquisition time of 4:57 min. 2D T2-FLAIR images were obtained with the following parameters: TR = 8000 ms, TE = 120 ms, TI = 2000 ms, acquisition matrix = 256x128, FOV = 210x210 mm^2^. We acquired 64 slices with a slice thickness of 2.5 mm in a total acquisition time of 3:13 min. DTI data were acquired in the axial plane with a single shot echo-planar imaging (EPI) sequence with 25 non-collinear directions and the following parameters: TR = 14200 ms, TE = 73.3 ms, flip angle = 90°, acquisition matrix = 128x64 (phase encoding, A-P direction), FOV = 220x220 mm2. Maximum b-value was 1000 s/mm^2^ and three volumes were acquired without diffusion weighting (b-value = 0 s/mm^2^). We acquired 70 contiguous slices with a slice thickness of 2.0 mm in a total acquisition time of 7:06 min. Whole brain functional MR images were obtained with a single shot T2* weighted EPI sequence sensitive to BOLD contrast with the following parameters: TR = 2000 ms, TE = 30 ms, flip angle = 75°, acquisition matrix = 64x96, FOV = 250x250 mm^2^. We acquired 26 contiguous slices with a slice thickness of 4.0 mm. The total acquisition time for the encoding and retrieval tasks were 7:20 min and 10:51 min respectively. Functional data acquisition started with 5 dummy scans, which were discarded from further analysis.

*fMRI task methodology and design*

Participants received instructions and practiced the task together with a researcher (JMP) 30 minutes prior to MRI scanning. During scanning, stimuli were visually presented outside the scanner using Presentation software (version 14.4, Neurobehavioral Systems Inc, Albany, CA, US), onto a back-projection screen that was visible with a mirror mounted on the head coil. Participants wore MRI-compatible goggles for correction of vision if needed. We presented stimuli with a fixed inter-stimulus interval of 4 s, and stimuli disappeared after a response, after which a black screen was presented until the next stimulus appeared. When participants did not respond within the inter-stimulus interval the next stimulus was presented upon termination of the inter-stimulus interval. External triggering by the MRI system ensured synchronization of the stimulus presentation and precise recording of task performance and response times. Participants responded with a left or right hand button press, which was recorded using MRI-compatible fibre optic response buttons. During the encoding phase we presented 52 mono- or bi-syllabic emotionally neutral Dutch words. We instructed participants to associate these words with either a positive feeling (right hand button press) or a negative feeling (left hand button press) to engage better encoding performance in line with Daselaar et al.[52]. We furthermore instructed participants to actively memorize the presented words during the encoding task. The emotionally neutral words in the encoding task were mixed with 52 baseline stimuli in a quasi-random way. Baseline stimuli consisted of the visual stimuli ‘left’ and ‘right’ to indicate respectively a left or right hand button press. During the retrieval phase, the same 52 words shown during the encoding task (‘target stimuli’) were randomly mixed with 52 new stimuli and 52 baseline stimuli. New stimuli consisted of words with the same lexical properties as the recognition stimuli. We instructed participants to indicate whether they recognized a stimulus from the encoding condition, by means of a right hand button press; or were confronted with a new stimulus by means of a left hand button press. The time between the encoding and retrieval task was about 10 minutes during which the participants performed a working memory n-back task, which is not further addressed in this paper [82].

*Functional MRI data preprocessing*

On an individual level, we spatially realigned all functional images using a rigid body transformation, and coregistered these images to the individual’s T1-weighted image. To normalize functional and anatomical images, we performed a unified segmentation/ normalization procedure to standard brain space defined by the Montreal Neurological Institute (MNI) as provided within SPM8. Functional images were resampled into 3x3x3 mm^3^ voxels and spatially smoothed with a 3D 6 mm full width half maximum Gaussian kernel. We calculated individual statistical parametric maps using the general linear model. Our design matrices included a high-pass filter of 128 s and 6 individual movement parameters to account for residual effects of head movement.**SUPPLEMENTARY RESULTS**

| **Supplementary Table 1** Within group activation and deactivation in MCI patients during successful episodic memory encoding and recognition | | | | | |
| --- | --- | --- | --- | --- | --- |
|  | Cluster size | X | Y | Z | T-value |
|  |  |  |  |  |  |
| **Activation successful encoding** |  |  |  |  |  |
| Medial frontal gyrus L | 604 | -6 | 59 | 25 | 8.81 |
| Cingulate gyrus L |  | -5 | 14 | 43 | 5.78 |
| Inferior frontal gyrus L | 974 | -45 | 23 | 10 | 7.72 |
| Postcentral gyrus L | 122 | -36 | -22 | 49 | 7.46 |
| Inferior occipital gyrus R | 126 | -36 | -88 | -8 | 7.38 |
| Inferior frontal gyrus R | 78 | 33 | 29 | 1 | 5.07 |
| Inferior occipital gyrus L | 77 | -27 | -94 | -8 | 4.96 |
| Middle temporal gyrus L | 20 | -45 | -70 | 25 | 4.27 |
| Middle temporal gyrus L | 24 | -51 | -40 | -2 | 4.17 |
| Posterior cingulate gyrus L | 20 | -3 | -55 | 10 | 4.00 |
| **Activation correct recognition** |  |  |  |  |  |
| Inferior frontal gyrus R | 4351 | 33 | 26 | 4 | 11.78 |
| Insula L |  | -30 | 26 | 1 | 11.53 |
| Inferior occipital gyrus R | 192 | 24 | -94 | -5 | 8.08 |
| Middle occipital gyrus R |  | 33 | -88 | 1 | 7.15 |
| Inferior occipital gyrus R |  | 42 | -85 | -5 | 5.99 |
| Angular gyrus L | 346 | -39 | -73 | 37 | 7.77 |
| Inferior parietal gyrus L |  | -45 | -58 | 49 | 7.70 |
| Middle occipital gyrus L | 32 | -30 | -94 | -5 | 5.65 |
| Inferior parietal gyrus R | 68 | 45 | -61 | 37 | 4.75 |
| Superior parietal gyrus R |  | 35 | -58 | 49 | 4.42 |
| Inferior parietal gyrus R |  | 42 | -70 | 40 | 4.09 |
| **Deactivation Successful encoding** |  |  |  |  |  |
| Postcentral gyrus R | 943 | 57 | -25 | 25 | 7.22 |
| Postcentral gyrus R |  | 45 | -37 | 49 | 6.21 |
| Inferior parietal gyrus R |  | 60 | -34 | 31 | 6.07 |
| Precuneus R | 200 | 6 | -55 | 40 | 5.63 |
| Posterior cingulate gyrus R |  | 9 | -46 | 28 | 4.85 |
| Mid cingulate gyrus R | 52 | 6 | -25 | 37 | 5.52 |
| Inferior parietal gyrus L | 50 | -60 | -49 | 40 | 5.36 |
| **Deactivation correct recognition** |  |  |  |  |  |
| Middle temporal gyrus R | 118 | 51 | -49 | 4 | 4.05 |
| Threshold p<0.001 uncorrected. L: left; R: right |  |  |  |  |  |

| **Supplementary Table 2** Within group activation and deactivation in MCI patients during successful episodic memory encoding and recognition corrected for MMSE | | | | | |
| --- | --- | --- | --- | --- | --- |
|  | Cluster size | X | Y | Z | T-value |
| **Activation successful encoding** |  |  |  |  |  |
| Medial frontal gyrus L | 602 | -6 | 59 | 25 | 8.99 |
| Cingulate gyrus L |  | -6 | 14 | 43 | 6.65 |
| Inferior frontal gyrus L | 971 | -45 | 23 | 10 | 8.05 |
| Postcentral gyrus L | 119 | -36 | -22 | 49 | 7.51 |
| Inferior occipital gyrus R | 131 | -36 | -88 | -8 | 7.30 |
| Inferior frontal gyrus R | 107 | 33 | 29 | 1 | 5.02 |
| Inferior occipital gyrus L | 80 | -27 | -94 | -8 | 4.97 |
| Middle temporal gyrus L | 25 | -51 | -40 | -2 | 4.23 |
| Posterior Cingulate gyrus L | 26 | -3 | -55 | 10 | 3.92 |
| **Activation correct recognition** |  |  |  |  |  |
| Inferior frontal gyrus R | 4429 | 33 | 26 | 4 | 11.78 |
| Insula L |  | -30 | 26 | 1 | 11.41 |
| Middle occipital gyrus R | 183 | 33 | -88 | 1 | 7.02 |
| Precuneus L | 328 | -42 | -73 | 37 | 7.66 |
| Middle occipital gyrus L | 30 | -30 | -94 | -5 | 5.55 |
| Inferior parietal gyrus R | 60 | 45 | -61 | 37 | 4.65 |
| **Deactivation successful encoding** |  |  |  |  |  |
| Postcentral gyrus R | 537 | 57 | -25 | 25 | 7.15 |
| Inferior parietal gyrus R |  | 60 | -34 | 31 | 6.08 |
| Superior temporal gyrus R |  | 54 | -49 | 16 | 6.04 |
| Postcentral gyrus R | 112 | 48 | -34 | 49 | 6.43 |
| Inferior parietal gyrus R |  | 45 | -52 | 52 | 3.28 |
| Precuneus R | 105 | 6 | -55 | 40 | 5.53 |
| Posterior cingulate gyrus R |  | 9 | -46 | 28 | 3.99 |
| Mid cingulate gyrus R | 32 | 6 | -25 | 37 | 5.52 |
| Inferior parietal gyrus L | 35 | -60 | -49 | 40 | 5.39 |
| **Deactivation correct recognition** |  |  |  |  |  |
| Middle temporal gyrus R | 25 | 54 | -52 | -2 | 4.46 |
| Threshold p<0.001 uncorrected. L: left; R: right |  |  |  |  |  |
